# Reactive Astrocytes as Drug Target in Alzheimer's Disease

**DOI:** 10.1155/2018/4160247

**Published:** 2018-05-14

**Authors:** Brhane Teklebrhan Assefa, Abadi Kahsu Gebre, Birhanetensay Masresha Altaye

**Affiliations:** Department of Pharmacology and Toxicology, School of Pharmacy, Mekelle University, Mekelle, Ethiopia

## Abstract

Alzheimer's disease is a neurodegenerative disease characterized by deposition of extracellular amyloid-*β*, intracellular neurofibrillary tangles, and loss of cortical neurons. However, the mechanism underlying neurodegeneration in Alzheimer's disease (AD) remains to be explored. Many of the researches on AD have been primarily focused on neuronal changes. Current research, however, broadens to give emphasis on the importance of nonneuronal cells, such as astrocytes. Astrocytes play fundamental roles in several cerebral functions and their dysfunctions promote neurodegeneration and, eventually, retraction of neuronal synapses, which leads to cognitive deficits found in AD. Astrocytes become reactive as a result of deposition of A*β*, which in turn have detrimental consequences, including decreased glutamate uptake due to reduced expression of uptake transporters, altered energy metabolism, altered ion homeostasis (K^+^ and Ca^+^), increased tonic inhibition, and increased release of cytokines and inflammatory mediators. In this review, recent insights on the involvement of, tonic inhibition, astrocytic glutamate transporters and aquaporin in the pathogenesis of Alzheimer's disease are provided. Compounds which increase expression of GLT1 have showed efficacy for AD in preclinical studies. Tonic inhibition mediated by GABA could also be a promising target and drugs that block the GABA synthesizing enzyme, MAO-B, have shown efficacy. However, there are contradictory evidences on the role of AQP4 in AD.

## 1. Alzheimer's Disease

Alzheimer's disease is a neurodegenerative disease clinically characterized by progressive deterioration of memory. In addition, histopathological changes such as deposition of extracellular amyloid-*β* (A*β*), intracellular neurofibrillary tangles (NFT) of hyperphosphorylated tau, and cortical neuron loss are widely noted [1, 2]. AD is the commonest form of dementia threatening 35.6 million people worldwide and this figure is expected to double every 20 years [3].

The exact mechanism behind AD development and progression is still unclear [4]. Several hypotheses, however, have been proposed to address the pathological lesions and neuronal cytopathology of the disease. Of these hypotheses, the amyloid metabolic cascade and the intracellular neurofibrillary tangles are considered the most important hypotheses [5, 6]. However, many pharmacological treatments targeting at these and other hypotheses have been unsuccessful to delay the progress of the disease significantly. This explains that no single theory alone is sufficient to explain the biochemical and pathological abnormalities of AD, which is believed to involve a multitude of cellular and biochemical changes [6, 7].

AD is characterized by the involvement of different cell types including activated astrocytes and microglia, characterized by gliosis and neuroinflammation, which in turn contributes to the neuronal dysfunction and death observed in AD [8]. Since AD pathologies are the result of neuronal death, search for mechanisms and therapeutic approaches have been neurocentric till a recent time [9]. However, the importance of nonneuronal cells, such as astrocytes, is now largely acknowledged and opened new research avenues that aim at better understanding of the pathology of the disease as well as characterizing new cellular and molecular targets for drug development [10, 11]. Thus the purpose of this review is to explore the role of reactive astrocytes in AD.

## 2. Astrocytes

Astrocytes are the most abundant cells in the brain and they can be broadly categorized into white matter astrocytes, gray matter astrocytes, ependymal astrocytes, radial glia, and perivascular astrocytes based on their anatomical location [12]. Astrocytes play a fundamental role in several cerebral functions such as the development and maintenance of blood brain barrier [13], the promotion of neurovascular coupling [14], the attraction of cells through the release of chemokines [14], K+ buffering [15], maintenance of general metabolism [16], control of the brain pH [16], uptake of glutamate and GABA by specific transporters [17], and production of antioxidants [18]. They are also involved in synaptogenesis and development of neuronal circuits by facilitating release of gliotransmitters [19]. This shows the paramount role of astrocytes interaction with neurons in process and control of synaptic formation [20]. Neuronal excitatory inputs activate astrocytes, which in turn mobilize Ca2+ resulting in gliotransmitters release including glutamate into synaptic cleft. The released glutamate increases neuronal excitability and modulates synaptic function [21, 22]. In addition, astrocytes are involved in uptake of glutamate from synaptic space by excitatory amino acid transporter including GLT1. Once uptaken, glutamate is metabolized into glutamine by glutamine synthetase before it is transferred to presynaptic neuron whereby glutamate and glutamine cycle is completed [23]. This is very important means of maintaining hemostasis of glutamate in the tripartite synapse and preventing glutamate induced excitotoxicity [24, 25]. Astrocytes also regulate GABA level as they are endowed with enzymes responsible for GABA synthesis (GAD 67) and metabolism (GABA-T) [26]. It also expresses reuptake transporter protein called GABA transporter protein thereby regulating the level of GABA at synapse [27]. AQP4 is also expressed at astrocyte endfoot processes and regulates cerebral water homeostasis. Recent studies also show the role of AQP4 in synaptic plasticity as reviewed by Szu and Binder (2015) [28].

## 3. Astrocyte Reactivity

Astrocytic reactivity is functional and morphological change of astrocytes as a result of variety of brain insults and it is characterized by increased gene expression of a number of astrocyte structural proteins, such as glial fibrillary acid protein (GFAP) and vimentin; morphological changes, such as hypertrophy of the cell soma and processing; and proliferation, which is particularly important in the formation of an astrocyte scar around tissue lesions [29]. It is usually implicated in several neurological disorders such as AD, Parkinson's disease, amyotrophic lateral sclerosis, Huntington's disease, and multiple sclerosis. Sustained reactive responses might be driven by positive feedback loops between microglia and astrocytes under conditions of severe and prolonged brain insults, thus providing detrimental signals that can compromise astrocytic and neuronal functions and lead to chronic neuroinflammation [30].

In patients with AD, reactive astrocytes are integral components of neuritic plaques and it seems to be particularly prominent around A*β* deposits both in the brain parenchyma and in the cerebrovasculature [31, 32]. However, their association with AD biomarkers and the functional impact of these cells and their therapeutic potential has remained elusive. Recent advances in cell-type-specific gene delivery techniques have helped hugely to identify unique beneficial and detrimental roles of astrocytes in neurodegenerative disorders suggesting that astrocytic signaling cascades can be selectively exploited for treating AD [33]. Detrimental effects of reactive astrocytes include altered glutamate homeostasis (decreased glutamate uptake due to reduced expression of uptake channel), altered energy metabolism, altered ion homeostasis (K^+^ and Ca^+^), increased glutamate, GABA, cytokines, and inflammatory mediators release [32]. Therefore, targeting specific astrocyte functions or specific aspects of reactive astrogliosis by targeting astrocyte-related molecular mechanisms is a viable option to counteract many CNS diseases. In this review, the altered gliotransmitters release (tonic GABA inhibition), altered glutamate metabolism, and aquaporins in reactive astrocytes as therapeutic target in Alzheimer disease are discussed.

## 4. Reactive Astrocytes as a Potential Target for Alzheimer's Disease

### 4.1. Altered Glutamate Homeostasis and Alzheimer's Disease

Glutamate, the major excitatory neurotransmitter, is carefully regulated by both neuronal and glial influences [34]. Astrocyte transports the vast majority of extracellular glutamate via excitatory amino acid transporters (EAATs). Of the five subtypes (EAAT1–EAAT5), EAAT2 (glutamate transporter-1/GLT1) is highly expressed throughout the brain and spinal cord and is responsible for more than 90% of total glutamate uptake [31]. In astrocytes, glutamate is converted to glutamine by an enzyme glutamine synthetase which then is shuttled back to presynaptic terminals and is used for the synthesis of the neurotransmitter glutamate. This process is called glutamate–glutamine shuttle and helps for keeping glutamate hemostasis in the brain [35]. Astrocytes damage in a way that affects their ability to sense or respond to an increase in glutamate levels, therefore, disrupts the microenvironment nearby neurons and it causes overstimulation of the NMDA receptors, which are responsible for modulation of the cognitive functions in the frontal cortex [11].

Normal physiological aging process is associated with reduced NMDA receptors and their function is related to the physiological memory decline. But these receptors, which are reduced in number and function due to aging, become overactive in certain regions of the brain (prefrontal cortex, hippocampus) in order to compensate for the memory loss which their continuous activation might trigger a glutamatergic cortical overactivation leading to excitotoxic damage of neurons [36]. Accumulation of excess extracellular glutamate and subsequent overstimulation of glutamatergic NMDA receptors are thought to have numerous neurotoxic effects such as calcium homeostasis dysfunction, increased nitric oxide (NO) production, activation of proteases, increase in cytotoxic transcription factors, and increased free radicals [30, 37].

An abnormal glutamate stimulation resulting in synaptic dysfunction has been proposed as one of several mechanisms by which synapses are damaged in AD [37–39]. Evidence shows downregulation of GLT1 is correlated with the cognitive decline seen in AD [40]. This was corroborated with GLT1 knockdown mouse models of AD which showed exacerbated cognitive decline [35]. Moreover, several studies have shown that GLT1 expression level is reduced in AD [41–44]. Interestingly recent* in vitro* studies suggested that A*β* species are responsible for GLT1 reduction and mislocalization in astrocytes, which leads to a marked reduction in the rate of glutamate clearance from the extracellular space [45, 46]. Recent study by Hefendehl et al. (2016) showed that glutamate clearance rates and GLT expression level are reduced in the direct vicinity of amyloid plaques (at a distance of 40–60 *μ*m from the amyloid plaque edge). Impressively the authors observed that the closer to the edge of the plaque the lower GLT level [46]. This is consistent with reports of Horiuchi et al. (2015) which showed decreased GLT expression in astrocyte cultures treated with amyloid-*β* [47]. These results suggest that A*β* induced astropathy is responsible for the reduced expression of GLT1 in AD and partly explains the A*β* pathomechanism.

Moreover, studies have demonstrated the possible correlation between alterations of GLT1 expression with astrocytic reactivity. Astrocyte reactivity caused by mechanical lesion was found to promote clustering of GLT1 immunoreactivity and with reduced glutamate transport activity which might contribute to increased extracellular glutamate concentrations and excitotoxic cell damage [48]. This is consistent with the finding of Lu et al. (2016) who found reduced GLT1 expression in astrocytes expressing higher level of GFAP [49]. Given that NMDA receptors are overexpressed in certain brain areas of aging population [36], reduced functions of GLT1 and overexpression of NMDA may have an overlapping role in induction of excitotoxicity and have been implicated in the pathogenesis of AD [37, 50].

The expression of GLT1 is regulated by nuclear factor kappa B (NF-kB) and N-myc which both are involved in TNF mediated transcriptional repression of GLT1 [51]. N-myc was found to be overexpressed in AD brains with reactive astrocytes [52]. Hence, N-myc overexpression may be the underlying mechanism causing the reduced GLT1 levels seen in AD brains. Another recent study showed that GLT expression is also regulated at posttranscriptional level [53]. Therefore we have two pharmacological approaches to increase the expression of GLT: by increasing GLT1 promoter activation [54] and by GLT1 translation activation [55].

Accordingly, drugs targeting astrocytic glutamate transporters to enhance their expression and function represent potential target for neurodegenerative disorders associated with excitotoxicity. Many chemical compounds have been tested for this purpose and showed efficacy. A study done on diverse library of 1,040 FDA approved drugs and nutritionals has shown the capability of over 20 compounds to increase GLT1 protein expression by more than twofold compared to untreated controls [54]. Of those compounds, *β*-lactam antibiotics were overly represented and fifteen different *β*-lactam antibiotics, including penicillin and its derivatives, as well as cephalosporin antibiotics, were highly active in stimulating GLT1 protein expression as early as 48 h after drug treatment. The study also revealed the potential of ceftriaxone to increase both brain expression of GLT1 and functional activity [54]. Ceftriaxone mediated increased expression of GLT1 is possibly NF-*Κ*B mediated GLT1 promoter activation [56]. Ceftriaxone was found to improve spatial learning and memory in chronically cerebral hypoperfused rats suggesting role in AD [57]. Moreover, ceftriaxone decreased tau pathology and showed improvement in cognitive functions [58]. Other compounds such as ampicillin [54], estrogen [59], Riluzole [60], and insulin [61] were also found to increase GLT1 expression.

From the second strategy, a representative lead compound LDN/OSU-0212320, a synthetic series of pyridazine derivative, was recently reported to show favorable GLT1 upregulation [55]. Colton et al. (2010) executed high-throughput screenings to search for compounds that increase GLT1 translation. Sixteen classes of compounds were found to activate GLT1 translation [62]. After intensive studies of these compounds, a pyridazine-based series was selected for further investigation and LDN/OSU-0212320 was selected as lead compound [63]. Pharmacological characterization by Kong et al. (2014) showed that LDN/OSU-0212320 protects cultured neurons from glutamate-mediated excitotoxic injury and death, delays motor function decline, and extended lifespan in an animal model of amyotrophic lateral sclerosis via GLT1 activation [55]. This translational activation is more attractive strategy because (1) loss of GLT1 protein in AD patients is most likely due to abnormality at the posttranscriptional level [64], (2) greater selectivity may be achieved, and (3) rapid effect can be produced [55].

Given glutamate induced excitotoxicity is one of the relevant hypotheses in the pathogenesis of AD, the findings of increased activity of NMDA receptors in certain brain areas (hippocampus and prefrontal cortex) and decreased GLT1 expression in reactive astrocytes promote AD as both of them are associated with excitotoxicity. Memantine is the only drug targeting excitotoxicity so far by blocking NMDA receptor approved for AD. However, clinical results indicate that the drug has only temporarily limited effects [36]. This calls further study on the GLT1 expression enhancers (both experimental and clinical studies) to see the efficacy of these drugs on disease progression when given alone or in combination with the NMDA antagonists in Alzheimer model animals and humans. There are no clinical studies on these drugs with regard to their efficacy in AD. Riluzole is, however, FDA approved for the treatment of ALS and its mechanism is partly related to upregulation of GLT [55]. A large clinical trial was done recently to test ceftriaxone in ALS patients but was stopped because predetermined criteria for efficacy were not met [65]. Therefore, clinical studies are warranted to determine their efficacy in AD.

### 4.2. Excessive Tonic Inhibition and Alzheimer's Disease

In the past, studies on AD focused on acetylcholine and glutamate neurotransmission and showed marked dysfunction while there was less evidence on the involvement of GABAergic neurotransmission [66]. However, recent studies have shown the role of GABAergic neurotransmission in the pathological changes of AD [67]. GABA is synthesized in neurons that express glutamic acid decarboxylase (GAD) [2]. GABA via GABA_A_ mediates both phasic inhibition and tonic inhibition in synaptic and extra synaptic sites, respectively [68, 69]. In some brain regions, such as prefrontal cortex (PFC), GABA_B_ receptors are also known to play a role [70]. Emerging evidences show that tonic inhibition is particularly important regulator of neuronal activity and network dynamics in cortical circuits as reviewed by McQuail et al. (2015) [71]. Recent data highlight that GABA receptors that mediate tonic inhibition are altered in aged hippocampus and PFC. The changes are, however, opposite in these brain areas such that there is decreased inhibition in the hippocampus [72–75] and increased tonic inhibition in the PFC [76–78].

These evidences suggest that normalizing GABA signaling may be an attractive target for cognitive dysfunction. However, the divergent changes observed in GABA transmission among hippocampus and PFC pose a challenge for the development of pharmacotherapy. So it is imperative to search another safe strategy to alleviate the excessive tonic inhibition observed in the dentate granule of Alzheimer's patients [69, 79].

In the molecular layer of dentate gyrus, diseased astrocytes produce abundant amount of GABA which is then released to inhibit excitatory neurotransmission to dentate gyrus. Dentate gyrus is the gate way of the cortical inputs to the hippocampus and it is essential for recall and memory [68]. The released GABA also spills over synaptic cleft and activates GABA receptors at extrasynaptic area. This is thought to be responsible for GABA mediated tonic inhibition [69].

Reactive astrocytes have been found around *β* amyloid since the disease was first described [80] and dentate gyrus area has more A*β* plaque deposits compared to other hippocampal regions of the brain [2]. Recent studies reported the unusual roles of these reactive astrocytes in the brain, that is, release of an excess GABA that impairs hippocampal memory in AD [69, 79]. In APP/PS1 mice model reactive astrocytes were found to aberrantly and abundantly produce inhibitory gliotransmitter GABA. In the dentate gyrus of mouse models of AD, the released GABA reduces spike probability of granule cells by acting on presynaptic GABA receptors. However, glutamate and phasic release of GABA were found unchanged [69]. Similarly, high GABA content in reactive astrocytes in the dentate gyrus of (5xFAD) AD mouse model was reported. This was associated with increased tonic inhibition and memory deficit [79]. These reports suggest that excessive tonic inhibition in AD is associated with astrocyte reactivity which in turn is caused by amyloidosis. In addition to GABA, monoamine oxidase-B (MAO-B) was found to be altered on reactive astrocytes [69]. The enzyme was found upregulated in the postmortem brain of individuals with AD [81, 82]. Interestingly, Jo et al. (2014) found that reactive astrocytes in hippocampus express minimal level of GAD, which suggests that GABA is instead synthesized by MAO-B through putrescine degradation [69]. Many reports have shown that GABA may be formed from putrescine in the animal CNS [83].

In Alzheimer's mice model, administration of GABA receptor antagonist was shown to improve hippocampal long-term potentiation and memory suggesting that aberrant GABAergic inhibition impairs memory in patients with AD [2]. GABA production or release from reactive astrocytes was found to fully restore the impaired spike probability, synaptic plasticity, learning, and memory in the mice. MAO-B inhibitors were found to successfully remove the tonic GABA inhibition on dentate gyrus granule neurons, revealed by the restoration of presynaptic stimulation-induced spike probability in these cells [69, 79]. Importantly, reducing tonic inhibition in mice was found to rescue the impairment of long-term potentiation and memory deficit [69, 79]. These results and the above data in concert lend support to the idea that excessive tonic inhibition from the dentate gyrus granule causes the memory decline in AD. Accordingly, reducing the excessive tonic inhibition from reactive astrocytes would be a novel therapeutic target in AD. This can be done by either blocking GABA_A_ receptor or blocking the synthesis and/or release of GABA from reactive astrocytes. GABA_A_ receptor inverse agonist, L-655708, was found to ameliorate the working memory deficits in AD mice [79]; this strategy is nonselective in its action, blocking both tonic and phasic inhibition. Hence it is not good therapeutic strategy as it may lead to many unwanted effects like increased risk of seizure and disinhibition of glutamate neurotransmission leading to excitotoxicity [84]. Owing to their selectivity to the excessive tonic inhibition, inhibition of synthesis and release of GABA from reactive astrocytes seems a better therapeutic target. Furthermore, GAT3/4 inhibitor SNAP-5114 and MAO-B inhibitor drugs selegiline were also found to effectively reduce memory impairment in AD mice [79, 83].

However, study by Brawek et al. (2018) challenged the aforementioned argument that tonic inhibition is a cause for the memory decline in AD [85]. In this recent study, they found a bell-shaped dependence between amyloidosis and GABA accumulation in astrocytes in a mouse model of AD. Their work depicted that GABA accumulation in astrocytes occurred during early stages of healthy aging, amyloid deposition. During early amyloid deposition, the astrocytic hypertrophy and the increase in astrocytic GABA content are both induced by plaque-related factors. They hypothesized that the increased astrocytic GABA content and a concomitant increase in the tonic inhibition is adaptive response of the brain, to fight plaque-mediated neuronal hyperactivity. This argument agrees with previous study by Héja et al. (2012) which showed that astrocytes are capable of converting neuronal hyperexcitability into tonic inhibition of the neighboring neurons via direct coupling between astrocytic glutamate uptake and GABA release from astrocytes [86]. Thus blocking this beneficial response of astrocytes would rather accelerate neural network dysfunction [85]. These conflicting arguments warrant further study to establish the exact role of astrocytic tonic inhibition on synaptic plasticity and Alzheimer's disease.

### 4.3. Role of Aquaporins in Alzheimer's Disease

The brain lacks a conventional lymphatic system; however, it uses the cerebrospinal fluid (CSF) to play a role equivalent to the systemic lymphatic system. Previously it was believed that CSF is almost exclusively produced by choroid plexus. But it is now understood that influx from the pericapillary space into the CSF system, namely, the interstitial flow, has significant contribution as well [87]. Accumulating evidences have shown that this interstitial flow which is regulated by aquaporin-4 (AQP-4) plays a critical role in the clearance of *β*-amyloid [88, 89].

Aquaporins (AQPs) are water channel proteins on the plasma membrane that function in the control of cellular water content. At least 13 water channel proteins (AQP1–13) have been identified in multiple mammalian species and AQP4 is the principal member of this protein family in the CNS, and it is widely expressed at the borders between the brain and major water-containing compartments, including CNS-CSF interface, CNS–blood interface. It has a polarized distribution on the astrocytes with tenfold higher density in glial endfoot membranes than in other membrane domains [90].

Apart from controlling water movement, AQP4 is involved in various astrocytic functions related to neurological diseases, potassium uptake, glutamate homeostasis, astrocyte migration and glial scarring, neural signal transduction, proinflammatory factor secretion, astrocyte-to-astrocyte cell communication, and synaptic plasticity. This could partially explain the potential mechanism for AQP4 mediating various functions of astrocytes [89, 91].

AQP4 has been studied in various brain pathological conditions like epilepsy and brain edema. However, its role in Alzheimer's disease is still unclear. The A*β* cascade is thought to be crucial in the etiology of AD. An abnormal A*β* deposition, which is regarded as the pathologic hallmark of AD, is thought to result from an imbalance between A*β* production and clearance [92]. Evidences show that the deposition of A*β* in the most common type of AD (sporadic AD) is due to decreased clearance as opposed to increased production [93, 94]. Age is the greatest risk factor for the sporadic form of AD [94] and it is associated with drastic decline in the efficiency of A*β* clearance [95]. Microdialysis studies also showed a strong correlation between the age dependent decrease in A*β* clearance and increase in A*β* deposition in APP transgenic mice [96].

Although there are many hypothesized A*β* clearance mechanisms, interstitial fluid (ISF) bulk flow which is mediated by AQP4 is associated with clearance of substantial amount of the A*β* [97–99]. CSF enters the parenchyma along paravascular spaces that surround penetrating arteries, and brain interstitial fluid is cleared along paravenous drainage pathways. Animals that lack AQP4 in astrocytes exhibit slowed CSF influx through this system and results of approximately 70% reduction of interstitial solute clearance [97]. This finding suggests that the bulk fluid flow between these anatomical influx and efflux routes is supported by astrocytic water transport.

A*β* has been demonstrated to be transported along this route. This was evidenced by aqp4 knockout mice which showed a decrease in A*β* clearance by 55–65% [97]. Similarly, deletion of AQP4 has showed a tendency to aggravate brain A*β* accumulation, subsequently exacerbating cognitive dysfunction in 12-month-old APP/PS1 mice. They also found that genetic deletion of AQP4 reduces A*β* induced activation of cultured astrocytes, which is associated with a reduction in the uptake of A*β* [100]. Senile plaque containing transgenic mice also showed a decline in water influx comparable to aqp4 knockout mice [97, 98]. Interestingly transgenic mice with increased A*β* production but without senile plaque formation showed no decrease in interstitial water flow supporting the hypothesis that a decreased A*β* clearance plays critical role in the formation of senile plaque as compared to the normal controls [101]. Furthermore this clearance was found to be reduced in aged mice by around 40% as compared to the young mice. This was supported with human studies which showed an increased deposition of A*β* in the brain of subjects aged 40 and 50 years [102]. All the above evidences support the hypothesis that A*β* deposition in Alzheimer's disease is due to decreased clearance as opposed to increased production. This is particularly important for the sporadic form of AD [103].

Moreover, several lines of evidences show that AQP4 expression level and localization are altered in Alzheimer's disease although initially differences have been noted in the reports of expression levels. Interestingly these differences can be ascribed to the stage of the disease or age of the senile plaque. In spite of this fact increased expression of AQP4 was observed in patients with AD. Moftakhar et al. (2010) showed that AQP4 displayed extensive immune reactivity around blood vessels in the CSF and brain interfaces [104]. Hoshi et al. (2012) also found similar results in which they found increased expression of AQP4 in cortical sections of temporal lobes of patients with AD [105]. But these authors came up with better characterization of the temporal variations of the AQP expression. Accordingly, AQP4 expression was increased during the early deposition of A*β* but downregulated in the later stages of A*β* plaque deposition. These observations were corroborated with AD mouse model studies. Besides this Igarashi et al. (2014) showed that AQP4 downregulation appears to occur in a later stage of A*β* plaque formation [87]. Yang et al. (2011) also demonstrated that AQP4 expression was significantly increased in Tg-ArcSwe mice at 9 months of age compared with wild-type mice and this difference was attenuated at 16 months of age [106]. Interestingly these alterations are associated with reactive astrogliosis [107]. Another study by Zeppenfeld et al. (2017) in postmortem brains of AD showed that loss of AQP4 polarization is the factor that renders the aging brain vulnerable to the misaggregation of amyloid-*β*, in AD [108]. Previous studies also support the mislocalization AQP4 as key factor in the aggregation extracellular solutes [95, 103]. These results show that it is not just decreased expression of AQP4 that causes the decrease in clearance of A*β*, rather loss of polarized expression plays critical role.

Research works over the past few years indicated that tau protein can propagate aggregates between cells. Once an aggregate is formed, it can exit the cells of origin into the interstitial space in response to neuronal excitation and can cross into other cells in the vicinity to induce further aggregation [109–111]. Accumulating evidences suggest that, like A*β*, extracellular tau protein also exits the brain via the interstitial bulk flow [98, 112, 113]. Iliff et al. (2014) demonstrated that aqp4 knockout was found to exacerbate impairment of glymphatic clearance and promoted the development of neurofibrillary pathology and neurodegeneration in the posttraumatic brain as compared to wild-type mice [112]. This indicates astrocytic AQP4 plays a key role in the clearance of extracellular tau protein.

Apart from the clearance of extracellular toxic solutes, AQP4 plays a regulatory potential on the function of GLT1 with respect to synaptic plasticity and memory. Several lines of evidences show that AQP4 and GLT1 exist in association on astrocytes. Zeng et al. (2007) and Li et al. (2012) showed that both GLT expression level and glutamate clearance were reduced in cultured astrocytes from aqp4 knockout mice [114]. This indicates that deficiency of AQP4 leads to downregulation of GLT1 which in turn affects synaptic plasticity and memory. Furthermore, AQP4 deficiency-induced impairment of synaptic plasticity and hippocampal memory deficit can be reduced by using GLT1 expression upregulators such as ceftriaxone [50]. All the above evidences lend support to the idea that AQP4 is a molecular target for Alzheimer's disease.

On the contrary, evidences show role of AQP4 in proinflammatory features of astrocytes, which could be an aggravating factor in the AD pathology. Huang et al. (2011) showed upregulation of AQP4 starting at 10 days and found significantly positive correlation between AQP4 and BBB disruption [115]. Similarly, Li et al. (2011) have shown detrimental inflammatory role of AQP4. These authors found less severe clinical and tissue inflammation score after lipopolysaccharide injection in AQP4 knockout than wild-type animals [67]. They also found reduced production of the proinflammatory cytokines: TNF*α* and IL-6 in AQP4 knockout mice astrocyte cultures. Therefore, further research is needed to clarify the overall role of AQP4 in the AD pathophysiological process. This should be a premise to exploit AQP4 for therapeutic strategies of AD.

Despite the presence of evidences on the involvement of AQP4 in Alzheimer's disease, no specific drug is developed to target this molecule to date probably due to its poor druggability. The development of such agents would lead to progress in the treatment of Alzheimer's disease. Recent studies however showed that the expression and polarity of AQP4 can be increased by some small molecule drugs. Lee et al. (2013) demonstrated that adenosine signaling regulates AQP4 expression [116]. Laurent et al. (2016) indicated that inactivation of adenosine 2A receptors (A_2A_Rs) reduced the intracellular phosphorylated tau level effectively in a mouse model of tauopathy [117]. Zhao et al. (2017) also showed that genetic deletion of A_2A_Rs alleviates the impairment of AQP4 polarity and accumulation of tau protein [118]. The above evidences in concert suggest that decreased extracellular tau is related to the amelioration of the impairment of AQP4 localization and this is interesting as loss of AQP4 polarized expression is critical for the decrease in extracellular A*β* and tau protein clearance [112]. Previous studies by Doll'lgna et al. (2003) showed that selective A_2A_R in-activators such as ZM 241385 prevented the neuronal cell death caused by exposure of rat cultured cerebellar neurons to *β*-amyloid protein [119]. Recent study by Zhao et al. (2017) reported that ZM 241385 reduces tauopathy related to A_2A_R inactivation [118].

The mechanism of effect of A_2A_Rs on AQP4 perivascular polarity is not clearly understood. Nonetheless previous studies showed that phosphorylation of AQP4 by kinases such as PKA and PKC regulates its distribution [120]. Interestingly activation of A_2A_R plays an important role in cAMP-PKA and PKC signaling pathways [121, 122]. These evidences shed light on the mechanism of A_2A_Rs mediated regulation of AQP4 expression. Thus development of selective blockers of A_2A_R seems to be an interesting future strategy for the treatment of Alzheimer's disease although it needs further corroboration.

## 5. Conclusion

Despite remarkable improvements in the understanding of the pathogenesis of AD, clear and accurate evidence on the mechanism of the disease is still lacking. Therapeutics so far targets majorly the hypothesis, excitotoxicity, and the cholinergic hypothesis and is symptomatic and hardly effective. However, the accumulating evidences on the importance of nonneuronal cells, such as astrocytes, opened new research avenues that aim at better understanding of the pathology of the disease as well as characterizing new cellular and molecular targets for drug development. The growing evidence on the physiological role of astrocytes in maintaining normal brain function shows that their altered functions due to reactivity play a key role in the etiology of AD and specific proteins such as MAO-B, the glutamate transporters, and AQP4 play key role in either protection or producing harmful effect and provide reliable targets for the pathogenesis of AD. According to current evidences, there are controversial findings on the role of reactive astrocytes in AD. Hence, further studies are warranted to fully characterize the effects of reactive astrocytes in AD and to search compounds which can modify their function.
